# Revisiting Cognitive Deficits in Outpatients with Psychotic Disorders: A Transdiagnostic Comparison of Cognitive Performance While Accounting for Putative Confounding Factors

**DOI:** 10.3390/brainsci14050446

**Published:** 2024-04-29

**Authors:** Mathias Konstantin Kammerer, Ulrike Nowak, Tania M. Lincoln, Katarina Krkovic

**Affiliations:** 1 Clinical Psychology and Psychotherapy, Institute of Psychology, Faculty of Psychology and Movement Sciences, Universität Hamburg, 20146 Hamburg, Germany; mathias.konstantin.kammerer@uni-hamburg.de (M.K.K.); nowak@praxis-uhr.de (U.N.); tania.lincoln@uni-hamburg.de (T.M.L.); 2 Clinical Psychology and Psychotherapy for Children and Youth, Department of Psychology, Faculty of Human Sciences, University of Potsdam, 14476 Potsdam, Germany

**Keywords:** delusions, sleep, defeatist-beliefs, actigraphy, verbal learning

## Abstract

Recent research suggests that cognitive deficits in individuals with psychotic disorders could be overestimated because poor cognitive test performance is partly attributable to non-cognitive factors. To further test this, we included non-hospitalized individuals with psychotic disorders (PSY, *n* = 38), individuals with attenuated psychotic symptoms (*n* = 40), individuals with obsessive-compulsive disorders (*n* = 39), and healthy controls (*n* = 38). Relevant cognitive domains were assessed using the MATRICS Consensus Cognitive Battery. Putative confounding non-cognitive factors—heart rate, self-reported stress, negative affect, performance-related beliefs, and actigraphy-derived sleep—were assessed before cognitive testing. A multivariate analysis of covariance was calculated to examine group differences in cognitive performance while controlling for non-cognitive factors. PSY showed decreased test performance in graphomotor speed, attention, and verbal tasks compared to the other groups, whereas non-verbal/visual-spatial tasks were unimpaired. After accounting for non-cognitive factors, group differences diminished in verbal learning, whereas differences in the other domains remained significant. Against our hypotheses, the present findings indicate that some cognitive deficits in PSY cannot be attributed to momentary confounding factors.

## 1. Introduction

Deficits in cognitive functioning are well documented and considered to be one of the core features of psychosis [1]. Cognitive deficits are present in first-degree relatives of individuals with psychotic disorders [2], as well as in individuals at-risk for psychosis [3], and are therefore considered to be a central vulnerability factor for psychotic disorders, as emphasized in vulnerability-stress models [4]. Notably, cognitive deficits are also considered to be the leading cause of persisting functional and related disabilities in patients with psychotic disorders [5]. This highlights the need to further investigate and understand the determinants of cognitive impairments in these individuals.

Although decades of research suggest that cognitive deficits in individuals with psychotic disorders are both generalized and stable [6], there is great heterogeneity between studies [1], and a variety of non-cognitive factors such as stress, negative affect, and motivation, can affect cognitive performance [7]. In line with this, some researchers have speculated that cognitive deficits in psychosis may be partly explicable by confounding non-cognitive factors, and thus may be overestimated [8,9]. To confidently exclude an overestimation of cognitive deficits in psychotic disorders, it is necessary to examine cognitive test performance in individuals with psychotic disorders and community samples while taking into account putative confounding factors.

A first group of factors that have been shown to potentially compromise cognitive performance are stress-related arousal [10] and negative affect [11]. Although moderate arousal can have advantageous effects on cognitive performance [12], high levels of stress-induced arousal adversely affect memory [13] and executive functions [14]. Likewise, negative affective states (e.g., anxiety, sadness) during cognitive testing can have detrimental effects on performance [15]. Elevated levels of stress and negative affect are well documented in psychotic disorders [16,17], and first research already indicates that cognitive deficits in individuals with psychotic disorders could be partly attributed to levels of stress [18] and negative affect [19].

A further confounding factor could be maladaptive beliefs about one’s ability to perform (i.e., self-efficacy expectancy/performance beliefs, defeatist beliefs), as these have been found to affect cognitive performance in previous studies [20,21] and are prevalent in many individuals with psychotic disorders [22,23]. Indeed, Ventura et al. (2014) [24] found lower self-efficacy in patients with psychotic disorders to be related to cognitive deficits. In another study, Moritz et al. (2017) [25] even found performance-related fear to explain differences in cognitive performance between patients diagnosed with psychotic disorders and healthy individuals. Hence, these findings give rise to reasonable doubt about the inherence of cognitive deficits in psychosis.

Another major factor that influences cognitive performance is disrupted sleep. Studies in community samples have repeatedly shown that cognitive performance and associated cortical networks are highly susceptible to sleep disruption [26,27]. One study that has addressed this topic in psychosis found circadian rhythm disruption to predict cognitive performance in individuals with psychotic disorders [28]. Another recent study found self-reported sleep disturbances (i.e., insomnia, hypersomnia) to be related to cognitive impairments in individuals with psychotic disorders [29]. However, although the majority of individuals with psychotic disorders exhibit sleep and circadian rhythm disruption [30], sleep deficits have been neglected in studies that examined differences in cognitive performance between individuals with psychotic disorders and other groups.

Taken together, various non-cognitive factors could potentially affect cognitive performance in individuals with psychotic disorders. Until now, only a few studies have accounted for these factors while examining group differences in cognitive performance and have mostly focused on one or a few isolated factors. Considering the lack of research and the fact that most putative confounding factors are likely to interact with each other, it would be of importance to replicate previous findings while accounting for different factors concurrently. Therefore, this study investigated performance in different cognitive domains using a standardized cognitive test battery while accounting for stress, negative affect, performance-related beliefs, and actigraphy-derived sleep. To extend our knowledge about whether the proposed confounding effects are specific to psychosis, we assessed individuals with psychotic disorders and current persecutory delusions (PSY), those with attenuated psychotic symptoms (AS), clinical controls with obsessive-compulsive disorders (OCD), which are also known to exhibit impairments in various cognitive domains [31] and can show symptomatic overlap with psychotic disorders [32]) and healthy controls (HC). We expected (1) groups to differ in cognitive performance, with PSY showing the lowest test scores. We further expected (2) cognitive performance to be associated with the tested non-cognitive factors. Finally, we expected that (3) group differences in cognitive performance would diminish after accounting for non-cognitive factors. 

## 2. Methods

This study was part of a larger randomized, repeated-measures trial on stress and flexible learning. Sample size was calculated a priori using G*Power (Version 3.1.5), expecting a small to medium effect (*f* = 0.175) and a drop-out rate of 10%. The study protocol was approved by the local ethics committee (date of ethical approval was the 13 February 2018; approval code of the ethics committee: 2017_142).

### 2.1. Participants

Participants were recruited using leaflets distributed at outpatient health facilities. Furthermore, we recruited participants through websites and forums. The recruitment took place between April 2018 and March 2020. The sample consisted of 155 participants who were assigned to one of four groups according to their diagnostic status: 38 participants to PSY, 40 to AS, 39 to OCD, and 38 to HC. General eligibility criteria were: (1) an age between 18 and 55 years; (2) informed consent; (3) being fluent in German; and (4) a verbal intelligence quotient of at least 85 in the Multiple-Choice Word Test, version B (MWT-B, German version; [33]). General exclusion criteria were: (1) dementia or any other neurological illness; (2) pregnancy; (3) a diagnosis of a bipolar disorder, social phobia, or a substance use disorder during the previous six months; and (4) acute suicidal tendency. Inclusion criteria for the PSY group were: (1) a diagnosis of a psychotic disorder (including F20, F22, F23, F25, F28, and F29 diagnoses according to ICD-10; determined via the Structured Clinical Interview for Mental Disorders, SCID, German version; [34]) adhering to the Diagnostic and Statistical Manual of Mental Disorder, 5th edition (DSM-5; [35]); and (2) the presence of delusions to at least a mild degree (items P1 or P6 ≥ 3) on the Positive and Negative Syndrome Scale (PANSS; [36]). Inclusion criteria for the AS group were: (1) an elevated frequency of positive psychotic symptoms in the subscale for positive symptoms (scores > 8; which is above the average of a community sample, based on [37]) of the Community Assessment of Psychic Experiences (CAPE; [38]); and (2) no current or lifetime diagnosis of a psychotic disorder. The inclusion criteria for the OCD group were: (1) a current diagnosis of obsessive-compulsive disorder (according to SCID adhering to DSM-5) and (2) no comorbid or lifetime diagnosis of psychotic disorder. The inclusion criteria for the HC group were: (1) no present or past diagnosis of any mental disorder according to DSM-5; (2) scores lower than 8 in positive psychotic symptoms on the CAPE positive symptoms subscale; and (3) no first-degree relatives with psychotic disorders.

### 2.2. Procedure

All participants attended three appointments at the laboratories of the Universität Hamburg (Germany). The first two appointments took place on consecutive days, and the third appointment took place seven days after the first appointment. At appointment one, general eligibility and exclusion criteria were assessed, and clinical interviews and self-report questionnaires were administered. Between appointments one and three, actigraphy-derived sleep was assessed. At appointment two, levels of momentary stress (physiological and self-reported), negative affect, and performance-related beliefs, as well as cognitive performance, were assessed in a quiet room. Subsequently, participants were exposed to either stress or a control condition (for further details, see [39]). At appointment three, participants were exposed to the remaining condition (stress/control), returned the actigraph, and were compensated with 120 €. The present analyses are exclusively based on the data gathered at appointment two (and thus before the stress induction) and include measures of momentary stress, negative affect, performance-related beliefs, actigaphy-derived sleep the night before appointment two, and cognitive performance.

### 2.3. Interview-Based and Self-Report Measures Assessed at Appointment One

To assess socio-demographic information, participants were asked to indicate their age, sex, education, employment, family status, as well as nicotine, alcohol, and drug use.

The Positive and Negative Syndrome Scale (PANSS; [36]), a semi-structured interview, was used to assess positive symptoms, negative symptoms, and general psychopathology in order to verify the eligibility of participants for the PSY group. The PANSS is widely used and has demonstrated good reliability and validity [40].

Additionally, we administered other self-report questionnaires (i.e., quality of sleep, insomnia, negative symptoms, obsessive-compulsive symptoms, well-being, and emotion regulation) that were not part of the present analyses.

### 2.4. Non-Cognitive Measures Assessed before Cognitive Testing

Heart rate was assessed as a physiological indicator of arousal (i.e., stress). An electrocardiogram (ECG) was recorded with a NeXus-10 Mark II (MindMedia, Roermond-Herten, The Netherlands) at a sampling rate of 256 Hz. We assessed resting-state heart rate during an interval length of 5 min in which participants were asked to remain seated and breathe spontaneously. Raw ECG data was extracted using the software Biotrace+ V2014A. We used Kubios HRV Premium (version 3.3.1; Kubios Oy, Kuopio, Finland) to manually correct R-peaks detection and conduct heart rate and time- and frequency-domain analyses. Heart rate was operationalized as the mean number of heartbeats per minute during a 5 min interval.

Self-reported momentary stress (based on [41]) was assessed with two items (“I feel stressed by the situation” and “I feel helplessly exposed to the situation”) that were rated on an eleven-point Likert-scale (0 = not at all; 10 = very much so), which have been shown to reliably assess momentary stress in previous studies [42]. Momentary stress was operationalized as the mean score across the two items. Cronbach’s alpha was 0.747. 

Self-reported momentary negative affect (based on [43]) was assessed with four items (“I feel angry/embarrassed/sad/anxious”) that were rated on an eleven-point Likert-scale (0 = not at all; 10 = very much so) which have been shown to reliably assess momentary negative affect in previous studies [44]. The momentary negative affect was operationalized as the mean score across these items. Cronbach’s alpha was 0.843.

Performance-related beliefs were assessed using the Achievement Motives Scale-Revised (AMS-R; [45]). The AMS-R comprises 10 self-report items that measure hope of success (e.g., “I enjoy situations, in which I can make use of my abilities”) and fear of failure (e.g., “I feel uneasy to do something if I am not sure of succeeding”). The items were rated on a four-point Likert scale (1 = not at all; 4 = very much so). The AMS-R has shown good reliability and validity [45]. Both subscales were operationalized as the sum scores across the respective items. Cronbach’s alphas were 0.822 (hope of success) and 0.878 (fear of failure).

Sleep measures were assessed by using wrist-worn actigraphs (Actiwatch 2, Philips Respironics, Murrysvile, PA, USA) with data collection at 15 s epochs over the period of seven nights between appointments one and three. Three sleep variables were calculated for the night before appointment two for each participant in accordance with recommended guidelines [46]: (1) total sleep time, the time slept in minutes without nocturnal wake intervals; (2) sleep efficiency, the percentage of time spent asleep related to the time spent in bed; and (3) wake after sleep onset, the time spent awake during sleep intervals in minutes. Actigraphy has shown good reliability and concordance with polysomnography [47]. See Section S1 for further details on the actigraphy assessment procedure.

### 2.5. Cognitive Performance Is Assessed at Appointment Two

To assess cognitive performance, we administered the MATRICS Consensus Cognitive Battery (MCCB; [48]), which has been shown to have excellent reliability and validity [49]. The MCCB measures six cognitive domains using the following subtests (for further details, see Section S2): (1) Speed of processing: (a) graphomotor speed: Brief Assessment of Cognition in Schizophrenia Symbol Coding (BACS); (b) verbal fluency: Category Fluency, Animal Naming (CF-AN); (c) visual search speed: Trail Making Test, Part A (TMT-A); and (d) task-switching: Trail Making Test, Part B (TMT-B; this test was added to compliment the domain speed of processing, as task-switching (i.e., attentional flexibility) is known to be compromised in psychotic individuals (e.g., [50]); (2) Attention/Vigilance: Continuous Performance Test, Identical Pairs (CPT-IP); (3) Working memory: (a) spatial non-verbal: Wechsler Memory Scale 3rd Ed.-Spatial Span (WMS-III); and (b) verbal: Letter-Number-Span (LNS); (4) Verbal learning: Hopkin’s Verbal Learning Test, Revised (HVLT-R); (5) Visual learning: Brief Visuospatial Memory Test, Revised (BVMT-R); (6) Reasoning and problem-solving: Neuropsychological Assessment Battery, Mazes (NAB). We derived T-scores corrected for age and gender from the proprietary software (International MSCEIT Branch 4 Scoring program).

### 2.6. Statistical Analyses

Data were analyzed using IBM SPSS Statistics (version 23.0) and R (version 4.0.0). We used univariate analysis of variance (ANOVA) and chi-squared tests to examine group differences in demographics. Because data were missing in some variables due to technical issues, we conducted Little’s test [51] to determine if missing data were missing completely at random (MCAR). Due to the substantial loss of statistical power in the case of listwise deletion (1 − *β*: 0.58 vs. 0.86), we conducted multiple imputations (MI) with 10 imputations using the fully conditional specification (FCS) method with predictive mean matching (PMM) in SPSS. All MATRICS variables, age, and group were included as predictors in the imputation model. Inferential statistical analyses were performed for the multiply imputed datasets. For results of statistical analyses performed for the original dataset, see Section S3.

Prior to conducting the main analyses, we examined statistical assumptions, and unless stated otherwise, the necessary assumptions were met. To test for group differences (PSY, AS, OCD, and HC) in cognitive performance (Hypothesis 1), we employed a multivariate analysis of variance (MANOVA) in R. Pillar’s trace was used as the test statistic for the multivariate effect. A significant main effect was followed up by post-hoc group comparisons. To test for associations between cognitive performance and non-cognitive factors (Hypothesis 2), we calculated Pearson correlation coefficients between cognitive test scores and non-cognitive factors across groups in SPSS. To test whether group differences in cognitive performance would diminish after accounting for non-cognitive factors (Hypothesis 3), we employed multivariate analyses of (co-)variance (MANCOVA) and entered the non-cognitive factors as covariates in R. Because cognitive performance is generally affected by age [52], we added age as a covariate to the multivariate analyses. Pillar’s trace was used as the test statistic for the multivariate effect. A significant main effect was followed up by post-hoc group comparisons using Tukey’s honest significant difference (HSD) test with Bonferroni correction for multiple comparisons. We obtained pooled *p* values (*p_pooled_*) for MAN(C)OVA in R by following the steps described by Finch (2016) [53]. We interpreted effect sizes using partial eta squared (η^2^_partial_). 

## 3. Results

### 3.1. Sample Characteristics and Preliminary Analyses

Groups differed significantly in psychotic experiences (via CAPE), age, and employment (see Table 1). Mean values and standard deviations of cognitive performances in each domain per group of the non-imputed dataset are depicted in Table 2. Data were missing in heart rate (29%), sleep measures (26.5%), self-reported stress and negative affect (14.2%), CPT-IP (6.5%), TMT-B (6.5%), TMT-A (3.2%), performance-related beliefs (1.3%), and the other MATRICS domains (1.3%). Little’s test revealed that missing data were missing completely at random, χ^2^ = 321.61, df = 282, *p* = 0.052. See Section S4 for descriptive statistics of the non-cognitive factors.

### 3.2. Group Differences in Cognitive Performance (Hypothesis 1)

MANCOVA controlled for age and gender revealed a significant multivariate main effect of group on cognitive performance, *F*_(30, 426)_ = 1.97, *p_pooled_* = 0.002, η^2^_partial_ = 0.121, 95% CI [0.016, 0.119]. Groups differed significantly in BACS, CPT-IP, LNS, and HVLT-R. Post-hoc analyses revealed that PSY, OCD, and AS differed significantly from each other and from HC in these tests (Table 3).

### 3.3. Associations between Cognitive Performance and Non-Cognitive Factors (Hypothesis 2)

Correlation analyses revealed BVMT-R and sleep efficiency (*p_pooled_* < 0.05) to be positively associated and NAB and fear of failure (*p_pooled_* < 0.01) to be negatively associated in the combined sample (Table 4). It needs mentioning that these correlations did not remain significant after correction for multiple comparisons with a false discovery rate of 5%.

### 3.4. Group Differences in Cognitive Performance While Accounting for Non-Cognitive Factors (Hypothesis 3)

MANCOVA controlled for age, gender, and non-cognitive factors revealed a significant multivariate main effect of group on cognitive performance, *F*_(30, 402)_ = 1.64, *p_pooled_* = 0.020, η^2^_partial_ = 0.109, 95% CI [0.002, 0.101]. In comparison to the model controlling for age and gender only, group differences in BACS and LNS decreased but remained significant. Group differences in CPT-IP increased and remained significant. Group differences in HVLT-R decreased and lost significance. Post-hoc analyses revealed that scores for HC and OCD in BACS did not differ significantly from each other. In CPT-IP and LNS, all groups differed significantly from each other. In BACS, scores from PSY differed significantly from all the other groups, and scores from AS differed significantly from OCD and HC (Table 3).

## 4. Discussion

This study examined whether poor cognitive performance in individuals with psychotic disorders could be attributed to confounding non-cognitive factors such as stress, negative affect, performance-related beliefs, and sleep disruption. We expected individuals with psychotic disorders to exhibit poorer performances in cognitive tasks compared to the other groups and expected performance differences (in parts) to be attributable to confounding factors. Results revealed significant performance deficits in individuals with psychotic disorders in tests of speed of processing, attention, working memory, and verbal learning. Deficits in verbal learning (HVLT-R) in individuals with psychotic disorders diminished after controlling for non-cognitive factors.

In line with decades of research (for a review, see [6]), the individuals with psychotic disorders showed significantly decreased performance in several cognitive tasks (i.e., graphomotor speed, sustained attention, verbal working memory, and verbal learning) compared to the other groups. In the tests of visual search speed, task switching, spatial working memory, visual learning, and reasoning, however, no group differences were found. Hence, our results confirm specific cognitive performance deficits in PSY (esp. in verbal tests and sustained attention) but indicate non-verbal/visual-spatial domains to be comparatively less impaired in our sample. This accords with some studies that have shown that visual learning and memory are generally less compromised compared to other domains of psychosis [5]. Moreover, performance deficits in verbal learning in PSY diminished considerably after accounting for the non-cognitive factors. This indicates that verbal learning deficits in individuals with psychotic disorders may be largely explicable by confounding factors. Nevertheless, this finding warrants replication and a better understanding of its potential causes. As such, the use and type of learning strategies may be promising candidates, as it has been shown that individuals with psychotic symptoms differ from non-psychotic individuals in their learning strategies in verbal learning [54]. Additionally, a recent meta-analysis showed that differences in verbal learning between individuals with psychotic disorders and mood disorders were prominently exhibited during acute phases [55], which raises the necessity to assess cognitive performance in individuals with psychotic disorders in different phases of illness and with different clinical control groups.

Fear of failure and sleep efficiency were associated with cognitive test performance in the combined sample, though these associations did not survive correction for multiple comparisons. Interestingly, in contrast to previous studies (e.g., [18,19]), we did not find stress and negative affect to be associated with cognitive performance. Nonetheless, since our study examined a larger number of non-cognitive factors concurrently, it potentially offers further insight into the relationship between cognitive performance and confounding factors. In contrast to the momentary assessment of negative affect and stress (e.g., “In this very moment, how anxious do you feel?”), fear of failure and sleep efficiency seem relatively stable over time [56,57]. One could thus speculate that cognitive performance in our sample was less prone to being affected by momentary factors. This, however, would need to be replicated in future studies to draw any final conclusions. In future studies, other potentially relevant factors that could confound cognitive test performance and that are relatively stable over time should be considered. Among others, these factors include loneliness [58], circadian rhythm disruption [28], motivational deficits [59], levels of inactivity [60], negative self-schemas [61], cannabis use [62], and somatic complaints [63], which are all known to be prevalent in individuals with psychotic disorders and to potentially affect cognitive performances.

Finally, divergent results regarding group differences in cognitive tasks need to be discussed: Cognitive impairment has been shown to already be present in premorbid phases of psychotic disorders [64]. In contrast to that, the individuals with attenuated psychotic symptoms in this study showed relatively high cognitive performance scores compared to the individuals with psychotic disorders and the healthy controls. However, the individuals with attenuated psychotic symptoms showed high education levels, while the healthy controls showed relatively low cognitive performance scores overall, which could have limited the representability of both groups. Furthermore, while previous studies found group differences in several cognitive tasks to be explicable by confounding factors (working memory, episodic memory, verbal comprehension: [18]; divided attention, executive functioning: [25]), we found cognitive deficits only in a single domain (i.e., verbal learning) to be attributable to confounding factors. As samples of individuals with psychotic disorders are highly heterogeneous across studies, varying symptom severity could contribute to the rather heterogeneous findings. In fact, some studies considered only inpatients [25], others considered in- and outpatients collectively [18,19], and the present study only included outpatients during non-acute phases and participants from the community. Hence, it is conceivable that the impact of confounding factors on cognitive performance is somewhat more prominent in hospitalized patients, as they experience an overall higher degree of psychological strain. Further supporting this, in previous studies that examined inpatients and outpatients collectively, patients exhibited considerable higher general psychopathology (via PANSS; 32.2 in [18]; 39.6 in [19]) compared to our sample. Against this background and in view of our results, it seems reasonable to assume that distinguishable confounding effects depend on the severity of symptoms and functional disabilities.

### Strengths and Limitations

Some limitations need to be considered. Firstly, although we used well-established self-report items [41,43], our study would have benefited from assessing further aspects of momentary stress and negative affect, such as other physiological indicators of stress (e.g., salivary cortisol) or cognitive appraisal. Secondly, one could speculate that repeated assessments before, during, and after cognitive testing would capture additional confounding effects on different tasks and across the temporal course of testing. This seems particularly important as some non-cognitive factors (e.g., negative affect, stress) are known to fluctuate over time and with task difficulty [65]. Nevertheless, both limitations cannot fully explain the diverging findings because previous studies also used single, primarily self-report assessments and still found confounding factors to contribute to group differences in cognitive performance [18,19,24,25]. Additionally, as psychotropic medication could have impacted cognitive performance in PSY and OCD, this could pose another confounding factor and should be considered in future studies [62,66]. Finally, while the focus on individuals with prevalent persecutory delusions is a strength of this study in terms of understanding persecutory symptomatology, it also limits the comparability and generalizability of our findings to other forms of psychotic symptomatology (including schizotypal personality disorders). Along the same line, apart from OCD, other clinical control samples (e.g., major depressive disorders) could be relevant in this context [54,55].

## 5. Conclusions

Taken together, despite significant group differences in several cognitive domains, non-verbal/visual-spatial domains were relatively unaffected in the individuals with psychotic disorders, and only verbal learning deficits could be attributed to the non-cognitive factors. Thus, in this study, cognitive deficits in individuals with psychotic disorders appeared to be rather stable and not merely a byproduct of confounding factors. The associations between cognition and non-cognitive factors might give some indication that cognitive performance in non-hospitalized individuals is less likely to be affected by momentary factors (e.g., affective states). However, this should be taken with a grain of salt, as the correlations found here did not survive correction for multiple comparisons. To the best of our knowledge, this is the third study to account for potential confounding factors while examining group differences in cognitive performance in psychosis and the second study to include clinical controls in the process. Given the lack of research in this field and the vast diversity of assessment methods across studies (i.e., state vs. trait measures, inpatients vs. outpatients, clinical control groups vs. healthy controls only, acute phase vs. non-acute phase, composite scores vs. subscales, established questionnaires vs. single items, cognitive test batteries vs. single tasks), rigorously conducted replication studies are needed to pin down the “true” extent of cognitive performance deficits in psychosis.

## Figures and Tables

**Table 1:** Sample characteristics.

	1. PSY (*N* = 38)	2. AS (*N* = 40)	3. OCD (*N* = 39)	4. HC (*N* = 38)	F/χ^2^	*p*	Post Hoc
Age (Mean ± SD)	37.71 ± 9.61	30.53 ± 10.58	35.92 ± 10.96	36.32 ± 11.18	3.496	0.014	2 < 3, 4, 1
Gender male/female (*N*)	19/19	18/22	14/25	20/18	2.54	0.469	-
Education ^a^ (*N*)							
low	2	1	4	1	10.88	0.092	-
middle	21	13	21	15			
high	15	26	14	22			
Unemployed ^b^ (*N*)	18	7	15	5	15.11	0.002	4 < 2, 3, 1
CAPE (Mean ± SD)	20.22 ± 10.15	15.56 ± 6.06	9.71 ± 7.43	3.71 ± 2.48	38.71	<0.001	1 > 2, 3, 4
PANSS (Mean ± SD)							
Positive symptoms	16.66 ± 4.23						
Negative symptoms	10.82 ± 3.91						
General psychopathology	26.42 ± 6.66						

Note: PSY = individuals with psychotic disorder; AS = individuals with attenuated symptoms; OCD = individuals with obsessive-compulsive disorders; HC = healthy controls; CAPE = Community Assessment of Psychic Experiences; PANSS = Positive and Negative Syndrome Scale; ^a^ low = equivalent of low-level general certificate of secondary education (GCSE) or no formal qualification; middle = equivalent of advanced-level GCSE or vocational qualification; high = university degree or A-level equivalent; ^b^ including “homemakers” and “retired”.

**Table 2:** Means and standard deviations of cognitive performance across groups (non-imputed dataset).

	1. PSY (*N* = 33)	2. AS (*N* = 37)	3. OCD (*N* = 33)	4. HC (*N* = 31)
	*M* (SD)	*M* (SD)	*M* (SD)	*M* (SD)
Speed of processing				
BACS	36.88 (10.29)	47.32 (14.57)	45.55 (11.24)	44.29 (9.11)
CF-AN	43.30 (8.02)	48.03 (10.65)	49.64 (8.89)	46.23 (10.27)
TMT-A	48.03 (11.76)	50.76 (8.99)	51.15 (10.50)	46.48 (9.47)
TMT-B	44.25 (12.38)	49.46 (12.36)	49.38 (12.71)	47.37 (10.68)
Attention				
CPT-IP	41.97 (11.01)	48.81 (8.09)	50.58 (6.61)	44.65 (10.06)
Working memory				
WMS-III	47.55 (9.72)	52.03 (8.96)	51.00 (11.36)	46.68 (9.33)
LNS	43.76 (10.61)	49.65 (7.29)	49.33 (9.72)	47.45 (12.46)
Verbal learning				
HVLT-R	44.73 (9.04)	48.16 (8.36)	50.12 (9.31)	51.45 (7.90)
Visual learning				
BVMT-R	45.61 (9.56)	51.41 (9.47)	49.94 (10.33)	46.81 (9.41)
Reasoning				
NAB	44.09 (11.21)	47.27 (8.02)	44.45 (10.03)	44.58 (9.99)

Note: BACS = Brief Assessment of Cognition in Schizophrenia; CF-AN = Category Fluency, Animal Naming; TMT-A = Trail Making Test, Part A; TMT-B = Trail Making Test, Part B; CPT-IP = Continuous Performance Test, Identical Pairs; WMS-III = Wechsler Memory Scale 3rd Ed., Spatial Span; LNS = Letter-Number-Span; HVLT-R = Hopkin’s Verbal Learning Test, Revised; BVMT-R = Brief Visuospatial Memory Test, Revised; NAB = Neuropsychological Assessment Battery, Mazes. Shown scores are *T*-values.

**Table 3:** Analyses of (co-)variance of cognitive performance across groups from multiply imputed datasets.

	ANCOVA Group Controlled for Age and Gender				ANCOVA Group Controlled for Age, Gender, and Non-Cognitive Factors			
	*F*_(3, 149)_	η^2^_partial_	*p_pooled_*	Post Hoc	*F*_(3, 141)_	η^2^_partial_	*p_pooled_*	Post Hoc
*Speed of processing*								
BACS	**4.99**	**0.091**	**0.003**	**1 < 4 < 3 < 2**	**3.69**	**0.073**	**0.013**	**1 < 4 = 3 < 2**
CF-AN	2.67	0.051	0.050	-	2.62	0.053	0.054	-
TMT-A	1.24	0.024	0.299	-	1.06	0.022	0.369	-
TMT-B	2.18	0.042	0.093	-	1.28	0.027	0.283	-
*Attention*								
CPT-IP	**6.15**	**0.110**	**<0.001**	**1 < 4 < 2 < 3**	**6.63**	**0.124**	**<0.001**	**1 < 4 < 2 < 3**
*Working memory*								
WMS-III	2.37	0.046	0.073	-	2.09	0.043	0.104	-
LNS	**3.78**	**0.071**	**0.012**	**1 < 4 < 2 < 3**	**2.91**	**0.058**	**0.037**	**1 < 4 < 2 < 3**
*Verbal learning*								
HVLT-R	**4.05**	**0.075**	**0.008**	**1 < 2 < 3 < 4**	2.31	0.047	0.079	-
*Visual learning*								
BVMT-R	2.28	0.044	0.081	-	1.54	0.032	0.207	-
*Reasoning*								
NAB	0.85	0.017	0.467	-	0.54	0.011	0.656	-

Note: BACS = Brief Assessment of Cognition in Schizophrenia; CF-AN = Category Fluency, Animal Naming; TMT-A = Trail Making Test, Part A; TMT-B = Trail Making Test, Part B; CPT-IP = Continuous Performance Test, Identical Pairs; WMS-III = Wechsler Memory Scale 3rd Ed., Spatial Span; LNS = Letter-Number-Span; HVLT-R = Hopkin’s Verbal Learning Test, Revised; BVMT-R = Brief Visuospatial Memory Test, Revised; NAB = Neuropsychological Assessment Battery, Mazes; 1 = individuals with psychotic disorders; 2 = individuals with attenuated symptoms; 3 = individuals with obsessive-compulsive disorders; 4 = healthy controls. Bold indicates significance of *p* < 0.05.

**Table 4:** Pearson correlations between cognitive performance and non-cognitive factors in the combined sample from multiply imputed datasets (*N* = 155).

	HR	Stress	NA	HS	FF	TST	SE	WASO
BACS	−0.080	−0.018	−0.122	0.137	−0.027	0.036	0.106	−0.043
CF-AN	−0.045	−0.017	−0.029	−0.094	−0.054	−0.094	0.034	−0.053
TMT-A	−0.052	0.041	0.050	0.047	−0.013	0.058	0.114	0.131
TMT-B	−0.054	0.025	−0.087	0.151	−0.158	0.054	0.099	0.045
CPT-IP	−0.073	−0.015	−0.026	0.002	−0.045	−0.071	−0.075	0.079
WMS-III	−0.107	0.028	0.029	0.036	0.011	0.032	0.053	0.083
LNS	−0.136	−0.023	−0.115	0.036	−0.091	−0.030	0.055	−0.020
HVLT-R	−0.158	0.006	−0.114	0.023	−0.155	−0.008	0.056	−0.092
BVMT-R	−0.164	−0.065	−0.105	0.145	0.039	0.132	0.245 *	−0.128
NAB	−0.123	−0.148	−0.111	0.045	−0.253 **	−0.101	0.034	0.110

Note: HR = heart rate; Stress = subjective stress; NA = negative affect; HS = hope of success; FF = fear of failure; TST = total sleep time; SE = sleep efficiency; WASO = wake after sleep onset; BACS = Brief Assessment of Cognition in Schizophrenia; CF-AN = Category Fluency, Animal Naming; TMT-A = Trail Making Test, Part A; TMT-B = Trail Making Test, Part B; CPT-IP = Continuous Performance Test, Identical Pairs; WMS-III = Wechsler Memory Scale 3rd Ed., Spatial Span; LNS = Letter-Number-Span; HVLT-R = Hopkin’s Verbal Learning Test, Revised; BVMT-R = Brief Visuospatial Memory Test, Revised; NAB = Neuropsychological Assessment Battery, Mazes. * *p* < 0.05; ** *p* < 0.01.

## Data Availability

The original contributions presented in the study are included in the article, further inquiries can be directed to the corresponding author.

## Supplementary Materials

The following supporting information can be downloaded at: https://www.mdpi.com/article/10.3390/brainsci14050446/s1, Section S1: Actigraphy assessment procedure; Section S2: MATRICS Consensus Cognitive Battery domains and subtests; Table S1: Analyses of (co-)variance of cognitive performance across groups from the non-imputed dataset; Table S2: Characteristics of non-cognitive factors from the non-imputed dataset.
