# Supplementary material for: Revisiting Cognitive Deficits in Outpatients with Psychotic Disorders: A Transdiagnostic Comparison of Cognitive Performance While Accounting for Putative Confounding Factors
Source: Brain Sci. 2024 Apr 29;14(5):446. doi: 10.3390/brainsci14050446 (PMC11119287; doi:10.3390/brainsci14050446)
Supplement: Supplementary file 1 [file brainsci-14-00446-s001.zip › brainsci-2960976-supplementary.pdf]

Section S1

Actigraphy assessment procedure

Before actigraphy data collection, participants were instructed to press a button on the actigraph, whenever they went to sleep and were provided with a one-week paper-pencil sleep diary. We used the proprietary software *Philips Actiware 6* (Version 6.05) with default settings (medium threshold for wake phase identification, ten consecutive inactive minutes to define sleep onset) to analyze actigraphy data. Three independent raters (including the first author) manually revised putative sleep episodes (i.e., button pressed before and after sections of low activity and low light amplitudes) that were not determined accurately by the integrated algorithm in accordance with the provided sleep diaries. Sections with missing or faulty data for more than one hour were excluded. In addition, consensus meetings with all three raters determined whether epochs with ambiguous data were appropriate for inclusion.

Section S2

MATRICS Consensus Cognitive Battery domains and subtests

Speed of processing: (a) Brief Assessment of Cognition in Schizophrenia (BACS) – Symbol Coding, a timed paper-pencil test of graphomotor speed in which participants have to put in digits that correspond to symbols; (b) Category Fluency – Animal Naming (CF-AN), an oral test of verbal fluency in which participants have to name as many animals as they can in one minute; (c) Trail Making Test – Part A (TMT-A), a timed paper-pencil test of visual search speed in which participants have to draw a line to connect numbered circles in ascending order; and (d) Trail Making Test – Part B (TMT-B), a timed test of task-switching as in TMT-A with additional circles with letters and the task to connect the circles in ascending order alternating between numbers and letters (i.e., 1-A-2-B-3-C etc.);

Attention/Vigilance: Continuous Performance Test – Identical Pairs (CPT-IP), a computer-administered test of sustained attention in which participants have to press a response button to consecutive matching numbers;

Working memory: (a) Wechsler Memory Scale 3rd Ed. – Spatial Span (WMS-III), a nonverbal test of spatial working memory using a board on which 10 cubes are irregularly spaced and in which participants have to tap cubes in the same (or reverse) sequence as the test administrator; and (b) Letter-Number-Span (LNS), a verbal test of verbal working memory in which participants have to reorder strings of numbers and letters mentally and repeat them to the test administrator;

Verbal learning: Hopkin's Verbal Learning Test – Revised (HVLT-R), a verbal test in which participants are presented with a list of 12 words from three taxonomic categories and have to repeat as many as possible after each of three learning trials;

Visual learning: Brief Visuospatial Memory Test – Revised (BVMT-R), a paper-pencil test in which participants are presented with six geometric figures that they then have to reproduce from memory;

Reasoning and problem-solving: Neuropsychological Assessment Battery – Mazes (NAB), a timed paper-pencil test in which participants have to complete seven mazes of increasing difficulties.

Section S3

Table S3

Analyses of (co-)variance of cognitive performance across groups from the non-imputed dataset

	ANCOVA group controlled for age and gender				ANCOVA group controlled for age, gender, and non-cognitive factors			
	$F(3,128)$	$\eta^2_{partial}$	p	Post hoc	$F(3,48)$	$\eta^2_{partial}$	p	Post hoc
Speed of processing								
BACS	**4.900**	**.103**	**.003**	**1 < 4,3,2**	**2.945**	**.155**	**.042**	**1<3,2,4**
CF-AN	2.439	.054	.068	-	2.605	.140	.063	-
TMT-A	1.751	.039	.160	-	1.251	.073	.302	-
TMT-B	1.229	.028	.302	-	0.248	.015	.862	-
Attention								
CPT-IP	**5.585**	**.116**	**.001**	**1 < 2,3**	**5.477**	**.255**	**.003**	**1< 2,3**
Working memory								
WMS-III	1.587	.036	.196	-	0.859	.051	.469	-
LNS	1.684	.038	.174	-	0.383	.023	.765	-
Verbal learning								
HVLT-R	**3.722**	**.080**	**.013**	**1 < 3,4**	0.885	.052	.455	-
Visual learning								
BVMT-R	1.829	.041	.145	-	0.661	.040	.580	-
Reasoning								
NAB	0.309	.007	.819	-	0.123	.008	.946	-

Note: BACS = Brief Assessment of Cognition in Schizophrenia; CF-AN = Category Fluency – Animal Naming; TMT-A = Trail Making Test – Part A; TMT-B = Trail Making Test – Part B; CPT-IP = Continuous Performance Test – Identical Pairs; WMS-III = Wechsler Memory Scale 3rd Ed. – Spatial Span; LNS = Letter-Number-Span; HVLT-R = Hopkin's Verbal Learning Test – Revised; BVMT-R = Brief Visuospatial Memory Test – Revised; NAB = Neuropsychological Assessment Battery – Mazes; 1 = individuals with psychotic disorder; 2 = individuals with attenuated symptoms; 3 = individuals with obsessive-compulsive disorders; 4 = healthy controls.

Section S4

Table S4

Characteristics of non-cognitive factors from the non-imputed dataset

	PSY	AS	OCD	HC
	Mean ± SD	Mean ± SD	Mean ± SD	Mean ± SD
Heart rate	81.81 ± 11.50	77.40 ± 10.69	76.51 ± 11.38	77.11 ± 8.84
Stress	2.11 ± 1.90	1.29 ± 1.53	2.42 ± 2.22	0.48 ± 0.80
Negative affect	1.99 ± 1.98	0.74 ± 1.02	1.57 ± 1.99	0.09 ± 0.23
Hope of success	15.55 ± 2.11	17.10 ± 2.91	15.74 ± 3.02	17.38 ± 2.46
Fear of failure	13.71 ± 3.32	11.64 ± 3.26	13.92 ± 3.62	10.12 ± 3.20
Total sleep time	405.02 ± 118.64	426.70 ± 71.71	401.19 ± 111.87	400.57 ± 97.63
Sleep efficiency	84.27 ± 8.72	85.39 ± 6.92	85.67 ± 6.36	86.08 ± 7.30
Wake after sleep onset	43.12 ± 33.68	33.14 ± 19.25	33.59 ± 22.30	27.24 ± 14.45

Note: PSY = individuals with psychotic disorder; AS = individuals with attenuated symptoms; OCD = individuals with obsessive-compulsive disorders; HC = healthy controls;
